# Recent Concepts of Ovarian Carcinogenesis: Type I and Type II

**DOI:** 10.1155/2014/934261

**Published:** 2014-04-23

**Authors:** Masafumi Koshiyama, Noriomi Matsumura, Ikuo Konishi

**Affiliations:** Department of Gynecology and Obstetrics, Kyoto University Graduate School of Medicine, 54 Shogoin, Kawahara-cho, Sakyo-ku, Kyoto 606-8507, Japan

## Abstract

Type I ovarian tumors, where precursor lesions in the ovary have clearly been described, include endometrioid, clear cell, mucinous, low grade serous, and transitional cell carcinomas, while type II tumors, where such lesions have not been described clearly and tumors may develop *de novo* from the tubal and/or ovarian surface epithelium, comprise high grade serous carcinomas, undifferentiated carcinomas, and carcinosarcomas. The carcinogenesis of endometrioid and clear cell carcinoma (CCC) arising from endometriotic cysts is significantly influenced by the free iron concentration, which is associated with cancer development through the induction of persistent oxidative stress. A subset of mucinous carcinomas develop in association with ovarian teratomas; however, the majority of these tumors do not harbor any teratomatous component. Other theories of their origin include mucinous metaplasia of surface epithelial inclusions, endometriosis, and Brenner tumors. Low grade serous carcinomas are thought to evolve in a stepwise fashion from benign serous cystadenoma to a serous borderline tumor (SBT). With regard to high grade serous carcinoma, the serous tubal intraepithelial carcinomas (STICs) of the junction of the fallopian tube epithelium with the mesothelium of the tubal serosa, termed the “tubal peritoneal junction” (TPJ), undergo malignant transformation due to their location, and metastasize to the nearby ovary and surrounding pelvic peritoneum. Other theories of their origin include the ovarian hilum cells.

## 1. Introduction

Ovarian carcinoma is the most lethal gynecological malignancy. It is estimated that there will be >140000 deaths per year worldwide [1]. Although many surgical techniques and chemotherapies have been developed for ovarian carcinoma, the prognosis remains poor, with a five-year survival rate of 45% [2]. Although the prognosis is more favorable in patients with stage I/II tumors, the majority of patients present with advanced stage disease (III/IV).

Most ovarian carcinomas have been suggested to originate from the ovarian surface epithelium or postovulatory inclusion cysts formed after follicular rupture and repair [3, 4]. Factors associated with a lower risk of developing ovarian cancer include pregnancies, the use of oral contraceptives, breast feeding, tubal ligation, and hysterectomy [5]. As of these factors are all associated with reduced numbers of ovulations, and it is believed that an increased lifetime number of ovulations play a significant role in the development of ovarian cancer [6].

According to the incessant ovulation hypothesis, every ovulation creates a wound, and the surface ovarian epithelial cells are then repaired by increased proliferation. This may increase the likelihood for DNA damage and carcinogenic mutations [5]. However, this hypothesis is inconsistent with the observation that patients with polycystic ovarian syndrome who have decreased ovulatory cycles appear to have an increased ovarian cancer risk [7]. The increased risk of ovarian cancer after the use of infertility drugs supports the fact that stimulation of the ovarian surface epithelium with gonadotropins increases the risk of ovarian cancer development [3, 8, 9]. The hypothesis regarding the gonadotropin-based stimulation is supported by the fact that the ovarian cancer incidence increases after menopause, when the gonadotropin levels rise [3, 10, 11].

Ovarian carcinomas have been classified according to the different epithelia of the reproductive female tract. The tumors are divided into serous, mucinous, endometrioid, clear cell, and transitional cell carcinomas. It has traditionally been thought that ovarian carcinomas are derived from the ovarian surface epithelium and that subsequent metaplasia leads to the development of the various cell types (serous, mucinous, endometrioid, clear cell, and transitional) which constitute the morphological subtypes of ovarian epithelial carcinomas. However, new histopathological, molecular, and genetic studies have recently provided a better model for ovarian carcinogenesis, showing two broad categories, which are designated as type I, where precursor lesions in the ovary have clearly been described, and type II, where such lesions have not been described clearly and tumors may develop* de novo* from the tubal and/or ovarian surface epithelium [4, 12]. Type I tumors include low grade serous, mucinous, endometrioid, clear cell, and transitional cell carcinomas, while type II tumors comprise high grade serous carcinomas, undifferentiated carcinomas, and carcinosarcomas.

Type I tumors are suggested to behave in an indolent behavior and appear to form part of a morphological and molecular continuum starting with cystadenoma/adenofibroma benign tumors that subsequently develop towards atypical proliferative or borderline tumors and then finally towards invasive tumors. They are often confined to the ovary at the time of diagnosis, with a stable genome and without TP53 mutations, although somatic mutations are frequently detected in a number of genes [13]. Each morphological subtype exhibits a distinctive molecular profile characterized by mutations targeting specific cell signaling pathways. Even though clear cell carcinoma is listed as a type I tumor, it may actually belong to an intermediate category because of its mutations and behavior.

Type II tumors are suggested to be more aggressive, are found at advanced stage, and are genetically highly unstable; the majority have TP53 mutations, and almost half of the cases have mutation, hypermethylation, or dysfunction of breast cancer gene (BRCA) 1/2 [14–17]. Several lines of evidence now indicate that these tumors may originate from the epithelium of the fimbrial portion of the fallopian tube [18–21] and/or the ovarian surface epithelium.

## 2. Type I

### 2.1. Endometrioid Carcinoma and Clear Cell Carcinoma

The recent study followed a cohort of 6398 women with clinically documented endometrioma and evaluated the risk of ovarian cancer based on the varying time periods from diagnosis of endometriosis [22]. During the follow-up of up to 17 years, 46 (0.7%) incidental ovarian cancers were identified, translating into a standardized incidence ratios of 13.2. This risk increased with age, with an incidence ratio of 13.2 in patients over 50 years old. In the malignant transformation cases of endometriotic cyst, serial transvaginal ultrasonography (USG) examinations revealed an increase in its size [23]. A review of 29 studies published from 1973 to 2002 on the prevalence of endometriosis in epithelial ovarian cancers organized by location of disease examined the different histologic subtypes with endometriosis in the same ovary. It was found that there was a prevalence of 4.5% in serous, 1.4% in mucinous, 35.9% in clear cell, and 19% in endometrioid carcinomas [24]. There is increasing evidence that clear cell and endometrioid carcinomas can arise from endometriosis. The specific correlation between endometriosis and ovarian malignancy and their epidemiological patterns have been studied. For both endometriosis and ovarian carcinoma, increased risks were associated with infertility, early menarche, late menopause, and nulliparity, and the protective factors were tubal ligation, hysterectomy, the use of oral contraceptives, and pregnancy [25].

Molecular aberrations that are characteristics of inflammatory processes in endometriosis may contribute a number of survival and growth signals to the malignant transformation of the ovarian surface epithelium. Endometriosis at the ovary confers an imbalance in the cytokine milieu (interleukin-1, interleukin-6, and interleukin-8) inducing surges of immunomodulatory and growth-stimulating cytokines (e.g., tumor necrosis factor- (TNF-) alpha) similar to those observed in ovarian malignancy. Endometriosis also drastically changes the hormonal milieu and generates growth factor (such as insulin-like growth factor (IGF)) to which ovarian cancer cells have demonstrated dependency [24]. The propensity of endometriotic cells to expand clonally, as a result of intrinsic anomalies and advanced inflammation in endometriosis, generates a constitutive abundant flux of several stimulatory signals, which induces progressive transcriptional changes that drive sustained proliferation. This also increases the rate of DNA repair and the likelihood of accumulation of mutations in these cells.

Mutations of the phosphatase and tensin homolog deleted from chromosome 10 (PTEN) tumor suppressor are frequently found in ovarian endometrioid carcinomas [26]. The identification of PTEN mutations in endometriotic lesions (20.6%) adjacent to ovarian endometrioid (20%) and clear cell carcinomas (8.3%) supports the notion that endometriosis is a precursor lesion for endometrioid and clear cell carcinomas [27]. In a mouse model of endometrioid ovarian carcinoma, PTEN deletion on the background of oncogenic K-RAS activation within the ovarian surface epithelium gave rise to endometriotic-like precursor lesions which developed into invasive endometrioid ovarian carcinoma within seven to twelve weeks [28]. These results indicate that expression of oncogenic K-RAS and inactivation of the PTEN tumor suppressor gene is an early event in the development of endometrioid carcinoma. Pathologically, the coexistence of ovarian carcinoma and endometriosis is frequently observed, with the latter called “atypical endometriosis,” which is a putative precursor lesion including atypia of cell nucleus [29].

The overexpression of hepatocyte nuclear factor-1 (HNF-1) beta [30] and mutations of the ARID1A gene [31] are also found in some atypical endometriosis adjacent to the carcinoma. Furthermore, the histogenesis of endometrioid carcinoma could arise from endometriosis, which originates from HNF-1 beta-negative inclusion cyst cells. In contrast, the expression of HNF-1 beta could be associated with the late secretory or menstrual phase endometrial-endometriosis—clear cell carcinoma (CCC) lineage, which means that CCC could arise from the HNF-1 beta—positive epithelial cells associated with endometriosis [32]. HNF-1 beta promotes aerobic glycolysis, which may contribute to cell survival under hypoxic conditions. The overexpression of HNF-1 beta may also play a role in the occurrence of CCC in stressful environment [33].

It has recently been recognized that carcinogenesis of endometrioid and clear cell carcinomas arising from endometriotic cysts is significantly influenced by the microenvironment in which the tumor arises [34]. As the content of an endometriotic cyst includes highly concentrated old blood, the concentration of iron is markedly high in endometriotic cysts [35]. Free iron is associated with cancer development through the induction of persistent oxidative stress. The epithelial cells within the endometriotic cyst are exposed to extensive oxidative stress (reactive oxygen species (ROS)) and hypoxia, and, as a result, they are subjected to more cellular and DNA damage and have less efficient DNA repair [36, 37].

Ovarian clear cell carcinomas (OCCCs) are rare tumors in Europe (4%) [38] and the United States (5%) [39]; however, they are common in Japan (20%). OCCCs are commonly considered to be chemoresistant tumors. Even though OCCC is listed as a type I tumor, it may actually belong to an intermediate category. Using a microarray dataset of ovarian cancers, the OCCC signature comprising 437 genes was identified [40]. Such OCCC signature genes contain many oxidative stress-related genes, which are actually upregulated by epigenetic mechanism in OCCCs. Therefore, it is estimated that the OCCC signature is first induced by the stressful environment in the endometriotic cyst and then becomes fixed during the course of development of the OCCC. It has been shown that there is similarity in the gene expression profile between renal cell carcinomas (RCCCs) and OCCC [41]. Sorafenib, which has recently been approved for RCCC, also showed significant antitumor activity in both of the two OCCC patients [42] and may thus represent a novel therapeutic agent for OCCC.

Mutations in the beta-catenin gene, CTNNB1, which is involved in cell proliferation and the Wnt pathway, have been found in up to 30% of endometrioid carcinomas but are uncommon in other subtypes [43, 44]. This finding suggests that beta-catenin and dysregulation of the wnt pathway are important in the development of endometrioid carcinomas. Furthermore, somatic mutation of the PI3K gene (PIK3CA) has been reported in 20% of endometrioid and clear cell carcinomas [45]. A more recent study has reported that PIK3CA mutations occur only in high grade endometrioid or high grade CCCs [46]. Microsatellite instability is present in endometrioid carcinoma and CCC but is only rarely detected in serous and mucinous tumors [47, 48].

### 2.2. Mucinous Carcinoma

There are several theories accounting for the origin of ovarian mucinous carcinoma. It is well recognized that a subset of mucinous carcinomas can develop in association with ovarian teratomas; however, the majority of these tumors do not harbor any teratomatous component [49, 50]. Other theories regarding the origin of these tumors include mucinous metaplasia of surface epithelial inclusions, endometriosis, and Brenner tumors [23, 50]. However, it is exceedingly rare to find mucinous metaplasia of the ovarian surface epithelium or within the lining of cortical inclusion cysts [51, 52], and there have only been a few case reports of such findings [53, 54]. Mucinous carcinomas can develop from endometriosis; however, this observation appears to be relatively uncommon, except for müllerian endocervical mucinous or mixed (seromucinous) borderline tumors [55, 56].

The association between Brenner and mucinous tumors has been known [57–59]. Amplification of 12q14-21 in both a mucinous carcinoma and an associated Brenner tumor was reported recently [60]. Mucinous carcinomas (intestinal type) and Brenner tumors may share similar histogenesis from transitional cell (Walthard) nests at the tubal peritoneal junction (TPJ) [61, 62]. Small mucinous tumors are rarely diagnosed, possibly because they are thought to be Brenner tumors with foci of mucinous differentiation [62].

Regardless of the origin of ovarian mucinous carcinoma, morphological transitions from cystadenoma to a mucinous borderline tumor (MBT) to intraepithelial carcinoma and invasive carcinoma have been recognized for some time, and an increasing frequency of KRAS mutations at codons 12 and 13 has been reported in cystadenomas, MBTs and mucinous carcinomas, respectively [63–66]. Similar to low grade serous carcinomas, mucinous carcinoma and adjacent MBT and mucinous cystadenoma show the same KRAS mutation, supporting the hypothesis of the “adenoma-carcinoma sequence” [67, 68] and the view that mucinous carcinomas develop in a stepwise fashion from mucinous cystadenomas and MBTs. These findings suggest that KRAS mutation is an early occurrence in the pathogenesis of ovarian mucinous tumors. Unlike low grade serous carcinomas, BRAF mutations are not a feature of ovarian mucinous neoplasms of intestinal type.

Most primary ovarian mucinous carcinomas (and borderline tumors) are of the so-called intestinal (enteric or nonspecific) type, unilateral, and stage 1. Advanced stage neoplasms (stage 3 or 4) are extremely uncommon. It is now clear that ovarian mucinous neoplasms associated with pseudomyxoma peritonei are almost always of vermiform appendix origin [69, 70]. While many primary ovarian mucinous carcinomas contain goblet cells and even occasionally Paneth or neuroendocrine cells, the presence of goblet cells is not a prerequisite for an intestinal type mucinous tumor. In fact, many of these more closely resemble gastric or pancreaticobiliary (upper gastrointestinal) mucinous neoplasms [71].

A much more uncommon müllerian (endocervical) type of ovarian mucinous carcinoma and borderline tumor also exists [72, 73]. While borderline mucinous neoplasms of müllerian type have been well described, malignant müllerian mucinous tumors are extremely uncommon.

According to several studies, smoking has been found to be a risk factor associated with benign, borderline, and mucinous carcinomas [74–76]. It has been speculated that the relationship between cigarette smoking and the development of mucinous tumors could be due to the similarity of mucinous tumors to the gastrointestinal mucosa. The latter tumors, such as the stomach and pancreas carcinomas, have consistently been associated with cigarette smoking [75].

### 2.3. Low Grade Serous Carcinoma

The low grade serous carcinomas are genetically stable and are characterized by their low number of genetic mutations. Therefore, they develop slowly from well-recognized precursors and behave in an indolent fashion. They are much less common than high grade serous carcinomas and are thought to evolve in a stepwise fashion from benign serous cystadenoma to serous borderline tumors (SBTs) (also referred to as atypical proliferative serous tumor) and finally to low grade serous carcinoma. Some authors have also suggested that serous tumors with micropapillary architecture may represent an intermediate step between SBTs and low grade serous carcinomas [77–80]. KRAS mutations at codons 12 and 13 occur in one-third of SBTs [81] and in 33% of low grade serous carcinomas [82]. Similarly, BRAF mutations at codon 599 occur in 28% of SBTs and 30% of low grade serous carcinomas [81, 83, 84]. Mutations in ERBB2 occur in less than 5% of these tumors. Mutations of KRAS and BRAF are detected in both SBTs and cystadenoma epithelium adjacent to SBTs [85]. These findings suggest that mutations of KRAS and BRAF are very early events in tumorigenesis, preceding the development of SBTs. The KRAS, BRAF, and ERBB2 oncogenes are upstream regulators of mitogen-activated protein kinase (MAPK), and mutations in these genes result in constitutive activation of the MAPK signal transduction pathway, which in turn leads to uncontrolled cell proliferation [17].

In contrast to high grade serous carcinoma, p53 mutations are uncommon in low grade serous carcinoma and are identified in <10% of these tumors [86]. A methylation profile distinct from that of high grade serous carcinoma has been identified in epigenetic studies [87]. Low grade serous carcinomas have a DNA content and level of copy number alterations which more closely resembles SBTs than high grade serous carcinomas and are intermediate between the two [88, 89]. A recent study involving a whole exome analysis of low grade serous carcinomas of the ovary identified an average of only 10 somatic mutations per tumor [90, 91]. In contrast, high grade serous carcinomas are generally aneuploidy, with a high level of copy number alterations [89]. These carcinomas typically sustain 50–70 somatic mutations, with TP53 representing a clear driver mutation [92].

Several theories exist to explain the origin of serous tumors. The traditional concept has been that they were derived from ovarian epithelial inclusions formed by invaginated ovarian surface epithelium that has undergone müllerian metaplasia [52]. It has been postulated that the native ovarian surface mesothelium possesses the potential to transform into an epithelial or mesenchymal phenotype in response to signals such as those associated with ovulation. The exposure of the mesothelial cells of an inclusion cyst to the ovarian stromal microenvironment may result in transformation to müllerian epithelium. However, well-documented examples of a transition of these cysts to serous carcinomas are rare.

Another theory is that tumors may be derived from a secondary müllerian system, thought to represent embryological remnants of the proximal müllerian ducts, located within the ovarian hilum [93, 94]. These müllerian epithelial cysts form the serous tumors, and their proliferation induces subsequent obliteration of the adjacent ovarian parenchyma. However, SBTs are only rarely reported to occur in the ovarian hilum [95, 96]. There was a recent theory which suggested that low grade serous carcinoma may be derived from the fallopian tube. The theory suggests that shed tubal epithelial cells can implant on the ovarian surface epithelium, be taken into inclusion cysts, and transform serous neoplasms, while implants on other peritoneal surfaces may account for extraovarian endosalpingiosis and noninvasive tumor implants [97, 98]. It is thought that chronic inflammation may induce the proliferation of the tubal epithelium, from which clusters of cells can then shed and implant on the ovarian and peritoneal surfaces, resulting in SBTs, noninvasive implants, and endosalpingiosis.

## 3. Type II

### 3.1. High Grade Serous Carcinoma

These tumors are high grade from the start, evolve quickly, and are frequently found at an advanced stage. At the molecular level, high grade serous carcinomas show TP53 gene mutations in nearly 80% of cases [14–17] and a high Ki67 proliferation index (between 50% and 75%). Overexpression of HER2/neu is also found in 20–67%, AKT activation in 12–30% and inactivation of p16 in 15% of cases. In addition, the overexpression of the human leukocyte antigen-G (HLA-G) system is found in 61% of cases, and there is overexpression of apolipoprotein E (apoE) in 66% of cases, but these are rarely found in low grade serous carcinomas. Chromosomal rearrangements are far more common in these types of tumors, probably reflecting the high degree of associated gene instability. Currently, up to 10–15% of ovarian carcinomas are believed to be hereditary [99]. Mutations in the high penetrance gene, BRCA1 and BRCA2, are associated with 90% of hereditary ovarian carcinoma cases. The lifetime risk of developing ovarian cancer is approximately 40–50% for BRCA1 mutation carriers and 20–30% for BRCA2 mutation carriers. Inherited BRCA1 and BRCA2 mutations predispose females to high grade serous carcinoma of the ovary.

In 2001, Piek et al. found new transformations on tubal segments removed from females who had either BRCA mutations or a strong family history of ovarian carcinoma who underwent a risk-reducing bilateral salpingo-oophorectomy (BSO) [18]. Of 12 tubal specimens, six had areas of cellular dysplasia noted in the tubal epithelium and five additional specimens had hyperplastic lesions in the microscopic findings. These dysplastic and hyperplastic lesions resembled high grade serous carcinoma but without stromal invasion. These malignancies were found in the distal tube in 4–17% of females with BRCA mutations at the time of their risk-reducing surgery, 57% to 100% of which were located in the distal portion of the tubes [100–104]. Dysplastic lesions within the tubal epithelium are termed “serous tubal intraepithelial carcinomas (STIC).” In 2003, Piek et al. hypothesized that hereditary serous carcinomas might originate from the epithelium of the fallopian tube which has spilled onto the surface of the ovary or peritoneum [105].

A very early abnormality termed “secretory cell outgrowths” (SCOUTs) has recently been reported [106]. This consists of a succession of at least 30 almost exclusively secretory epithelial cells with a pseudostratified appearance [107]. An immunohistochemical analysis can confirm the diagnosis, which is characterized by a low PTEN and Ki67 index, and, in most cases, there are no TP53 mutations [106, 108–110]. The TP53 signatures are the next earlier entities, which have an immunohistochemical definition of at least 12 consecutive secretory cells that are p53 positive and have a low proliferative index (Ki67 < 10%). In the next place, “serous tubal intraepithelial lesions” (STILs) [111] also called “transitional intraepithelial lesions of the tube” (TILTs) by some authors have proliferative p53 signatures, tubal dysplasia, and even tubal epithelial atypia [18, 112]. These have also been described as a group of tubal anomalies with different p53 signatures compared to STICs [113]. In the serous carcinogenic sequence, SCOUTs may be able to evoke in benign lesions expressing p53 (p53 signature) with a low proliferation index and little genetic instability. Then, benign lesions expressing p53 would appear (STILs/TILTs), corresponding to tubal dysplasia lesions, finally culminating in the appearance of STICs [114]. Rate of the STICs was 59% in patients with serous tumors [21] and the former was clonally related to the latter [115]. There were no STICs identified in mucinous, endometrioid, or carcinosarcoma histology. Thus, STICs seem to be associated with the development of serous carcinoma.

It has recently been reported that the junction of the fallopian tube epithelium with the mesothelium of the tubal serosa, termed the “TPJ,” might be a potential site of carcinogenesis, as the role of epithelial junctions, notably the uterine cervical squamocolumnar, gastroesophageal, and anorectal junctions, in neoplasia is well recognized [116]. This junction is highly tortuous with tongues of mesothelium extending from the infundibular peritoneal-fimbrial junction at the outer edges of the fimbriae, with irregular tongues of peritoneum extending onto some of the plicae. The extensive and elaborate lymphovascular system is in almost direct contact with the basement membrane of the tubal epithelium, suggesting that even a minimally invasive carcinoma could easily invade this system and rapidly spread throughout the abdominal cavity.

Given that STICs have shorter telomeres than high grade serous ovarian carcinoma and also have gamma H2AX overexpression, these results seem to suggest that DNA repair mechanisms are activated in the early conditions [117]. Telomere shortening appears to take place in most human preinvasive epithelial lesions [118]. As a result, some authors have hypothesized that STICs are not metastases from ovarian carcinoma (different telomere lengths between STICs and ovarian carcinomas) but tubal precursor lesions of ovarian carcinoma [119]. In brief, the small areas of STICs undergo malignant transformation and, due to their location, metastasize to the nearby ovary and surrounding pelvic peritoneum.

On the other hand, serous carcinoma may have a truly ovarian origin. Our group reviewed the clinical charts of 543 patients with epithelial ovarian carcinomas who underwent laparotomy and collected patients whose clinical and transvaginal ultrasonography (USG) findings for adnexal regions 12 months or fewer prior to the surgery were available [23]. The data of 35 patients were available (11 serous, 6 mucinous, 8 clear cell, and 10 endometrioid). In their series of serous carcinomas, there had been no apparent abnormalities in the adnexal regions, 2 to 12 months prior to the diagnosis in 9 of the 11 (82%) patients. Strikingly, 8 of 9 serous carcinoma patients with no apparent abnormalities at the last visit presented with stage III tumors and the final pathological findings after surgery showed that there were no malignancies in adjacent lesions, indicating that serous carcinomas might develop from the ovaries suddenly and progress very rapidly. However, their report raises the possibility that there might be the small malignancy lesion of the fallopian tubes which was not able to be detected in some cases.

In 1994, Bell and Scully reported 14 cases of incidentally found microcarcinoma in normal-appearing ovary [120]. Most of them were serous carcinoma, suggesting* de novo* carcinogenesis, whereas there were no cases of mucinous carcinoma. Furthermore, a notable study has recently been reported, in which the ovarian hilum cells show increased transformation potential after inactivation of tumor suppressor genes transformation-related protein 53 (Trp53) and retinoblastoma 1 (Rb1) in mice [121]. These pathways are altered frequently in the high grade serous carcinoma. In brief, the ovarian surface epithelium (OSE) at the TPJ contains a novel stem cell niche that is responsible for OSE regeneration and is prone to malignant transformation. These stem cells in the hilum may have increased transformation potential after inactivation of Trp 53 and Rb 1 and be the origin of high grade serous carcinoma.

## 4. Conclusions

The new model of assigning ovarian epithelial carcinomas into two groups demands a radical change of current clinical management. Type I ovarian carcinomas are considered to arise via a well-defined adenoma-carcinoma sequence from a benign precursor lesion, such as a borderline tumor or endometriosis, and to evolve in a stepwise fashion. Type I carcinomas are, in general, slow growing, indolent neoplasms, and like type I endometrial carcinomas. In contrast, type II carcinomas are high grade clinically aggressive neoplasms. Most represent high grade serous carcinoma. Carcinosarcoma and undifferentiated carcinoma, which are both predominantly variants of high grade serous carcinoma, are also included in this category. Type II carcinomas are often associated with TP53 mutations and are like type II endometrial carcinomas. There is emerging evidence that many arise from the epithelium of the distal fallopian tube and/or ovary.

Clinicopathological features and molecular genetic alterations of two types of ovarian carcinoma are summarized in Table 1.

We study the carcinogenesis of ovarian carcinoma to determine the characteristics of each subtype carcinoma and to optimize the treatment of the disease. For example, there are similarities in the gene expression between RCCC and OCCC, as determined using a microarray analysis. Sorafenib, which has recently been approved for RCCC, also showed significant antitumor activity in the patients with recurrent chemoresistant OCCC. With regard to benign cysts of the ovary (e.g., endometriotic, serous, and mucinous cysts), there need to be a unification of the preventive treatment strategy and to detect an early cancer by careful follow-up with USG at every 6 months and some bioindices (countermeasures against type I carcinomas). Complete bilateral salpingo-oophorectomy as a risk-reducing strategy in patients with BRCA mutations in an approach is worthy of further investigation and it may be reasonable to consider bilateral salpingectomy for all patients undergoing hysterectomy for benign disease (countermeasures against type II carcinomas). In other words, we are entering a period of individualized therapies including preventive therapies, where it is necessary to know the characteristics of each carcinoma using biomarkers and gene profiling. We hope that all type I and type II carcinomas of the ovary will be able to be prevented and/or cured completely in the near future.

## Figures and Tables

**Table 1:** Summary of clinicopathological features and molecular genetic alterations of two types of ovarian carcinoma.

	Type I tumors		Type II tumors	
Behavior	Indolent		Aggressive	
At the time of the diagnosis	Early stage		Advanced stage	
Survival rate at 5 years	About 55%		About 30%	
Histological type/Precursors				
	Endometrioid carcinoma/	Endometriosis	High grade serous/	Probably *de novo* starting at the tubo-
	Clear cell carcinoma/	Endometriosis		ovarian surface epithelium; SCOUT→
	Mucinous carcinoma/	Mucinous Cystadenoma, Endometriosis		P53 signature→STIL/TILT→STIC
		Teratoma, Brenner Tumor, and MBT		or ovarian hilum stem cell
	Low grade serous carcinoma/	Serous cystadenoma, Adenofibroma	Undifferentiated carcinoma/	?
		Atypical proliferative serous tumor (SBT)	Carcinosarcoma/	?
		Müllerian epithelial cyst		
	Transitional cell carcinoma/	Brenner tumor		
Gene expression profile				
Genetic instability	Not very unstable		Very unstable	
PTEN mutation	15–20%		Low	
HNF-1 beta overexpression	90%		Low	
ARID1A mutation	40–50%		Not found	
CTNNB1 mutation	30%		Low	
PIK3CA	20%		Low	
Microsatellite instability	50%		8–28%	
KRAS mutation	30–65%		Low	
BRAF mutation	30–65%		Low	
TP53 mutation	Low		50–80%	
HER2/neu overexpression	Low		20–67%	
AKT overexpression	Low		12–30%	
p16 inactivation	Low		15%	
HLA-G overexpression	Low		61%	
APO E overexpression	12%		66%	
BRCA 1/BRCA2 mutation	Low		High	
Ki67 proliferation index	10–15%		50–75%	

## References

[1] Jemal A, Bray F, Center MM, Ferlay J, Ward E, Forman D (2011). Global cancer statistics. *CA: Cancer Journal for Clinicians*.

[2] Siegel R, Ward E, Brawley O, Jemal A (2011). Cancer statistics, 2011: the impact of eliminating socioeconomic and racial disparities on premature cancer deaths. *CA: Cancer Journal for Clinicians*.

[3] Mok SC, Kwong J, Welch WR (2007). Etiology and pathogenesis of epithelial ovarian cancer. *Disease Markers*.

[4] Landen CN, Birrer MJ, Sood AK (2008). Early events in the pathogenesis of epithelial ovarian cancer. *Journal of Clinical Oncology*.

[5] Tortolero-Luna G, Mitchell MF (1995). The epidemiology of ovarian cancer. *Journal of Cellular Biochemistry*.

[6] Ricciardelli C, Oehler MK (2009). Diverse molecular pathways in ovarian cancer and their clinical significance. *Maturitas*.

[7] Schildkraut JM, Schwingl PJ, Bastos E, Evanoff A, Hughes C, Curtin JP (1996). Epithelial ovarian cancer risk among women with polycystic ovary syndrome. *Obstetrics and Gynecology*.

[8] Komatsu T, Konishi I, Mandai M (1995). Peritoneal papillary serous carcinoma arising in an infertile woman during ovulation-induction therapy: immunohistochemical expression of LH/hCG receptors. *Gynecologic Oncology*.

[9] Kobayashi F, Monma C, Nanbu K, Konishi I, Sagawa N, Mori T (1996). Case report: rapid growth of an ovarian clear cell carcinoma expressing LH/hCG receptor arising from endometriosis during early pregnancy. *Gynecologic Oncology*.

[10] Kuroda H, Mandai M, Konishi I (2001). Human ovarian surface epithelial (OSE) cells express LH/hCG receptors, and hCG inhibits apoptosis of OSE cells via up-regulation of insulin-like growth factor-1. *International Journal of Cancer*.

[11] Mandai M, Konishi I, Kuroda H, Fujii S (2007). LH/hCG action and development of ovarian cancer—a short review on biological and clinical/epidemiological aspects. *Molecular and Cellular Endocrinology*.

[12] Shih IM, Kurman RJ (2004). Ovarian tumorigenesis: a proposed model based on morphological and molecular genetic analysis. *The American Journal of Pathology*.

[13] Kurman RJ, Shih IM (2011). Molecular pathogenesis and extraovarian origin of epithelial ovarian cancer—shifting the paradigm. *Human Pathology*.

[14] Koshiyama M, Konishi I, Mandai M (1995). Immunohistochemical analysis of p53 protein and 72 kDa heat shock protein (HSP72) expression in ovarian carcinomas: correlation with clinicopathology and sex steroid receptor status. *Virchows Archiv*.

[15] Santin AD, Zhan F, Bellone S (2004). Gene expression profiles in primary ovarian serous papillary tumors and normal ovarian epithelium: identification of candidate molecular markers for ovarian cancer diagnosis and therapy. *International Journal of Cancer*.

[16] Salani R, Kurman RJ, Giuntoli R (2008). Assessment of TP53 mutation using purified tissue samples of ovarian serous carcinomas reveals a higher mutation rate than previously reported and does not correlate with drug resistance. *International Journal of Gynecological Cancer*.

[17] Cho KR, Shih IM (2009). Ovarian cancer. *Annual Review of Pathology*.

[18] Piek JMJ, van Diest PJ, Zweemer RP (2001). Dysplastic changes in prophylactically removed fallopian tubes of women predisposed to developing ovarian cancer. *Journal of Pathology*.

[19] Piek JMJ, Kenemans P, Verheijen RHM (2004). Intraperitoneal serous adenocarcinoma: a critical appraisal of three hypotheses on its cause. *The American Journal of Obstetrics and Gynecology*.

[20] Maeda D, Ota S, Takazawa Y (2010). Mucosal carcinoma of the fallopian tube coexists with ovarian cancer of serous subtype only: a study of Japanese cases. *Virchows Archiv*.

[21] Przybycin CG, Kurman RJ, Ronnett BM, Shih I, Vang R (2010). Are all pelvic (nonuterine) serous carcinomas of tubal origin?. *The American Journal of Surgical Pathology*.

[22] Kobayashi H, Sumimoto K, Moniwa N (2007). Risk of developing ovarian cancer among women with ovarian endometrioma: a cohort study in Shizuoka, Japan. *International Journal of Gynecological Cancer*.

[23] Horiuchi A, Itoh K, Shimizu M (2003). Toward understanding the natural history of ovarian carcinoma development: a clinicopathological approach. *Gynecologic Oncology*.

[24] Nezhat F, Datta MS, Hanson V, Pejovic T, Nezhat C, Nezhat C (2008). The relationship of endometriosis and ovarian malignancy: a review. *Fertility and Sterility*.

[25] van Gorp T, Amant F, Neven P, Vergote I, Moerman P (2004). Endometriosis and the development of malignant tumours of the pelvis. A review of literature. *Best Practice and Research: Clinical Obstetrics and Gynaecology*.

[26] Obata K, Morland SJ, Watson RH (1998). Frequent PTEN/MMAC mutations in endometrioid but not serous or mucinous epithelial ovarian tumors. *Cancer Research*.

[27] Sato N, Tsunoda H, Nishida M (2000). Loss of heterozygosity on 10q23.3 and mutation of the tumor suppressor gene PTEN in benign endometrial cyst of the ovary: possible sequence progression from benign endometrial cyst to endometrioid carcinoma and clear cell carcinoma of the ovary. *Cancer Research*.

[28] Dinulescu DM, Ince TA, Quade BJ, Shafer SA, Crowley D, Jacks T (2005). Role of K-ras and Pten in the development of mouse models of endometriosis and endometrioid ovarian cancer. *Nature Medicine*.

[29] Mandai M, Yamaguchi K, Matsumura N, Baba T, Konishi I (2009). Ovarian cancer in endometriosis: molecular biology, pathology, and clinical management. *International Journal of Clinical Oncology*.

[30] Kato N, Sasou S, Motoyama T (2006). Expression of hepatocyte nuclear factor-1*β* (HNF-1*β*) in clear cell tumors and endometriosis of the ovary. *Modern Pathology*.

[31] Wiegand KC, Shah SP, Al-Agha OM (2010). ARID1A mutations in endometriosis-associated ovarian carcinomas. *The New England Journal of Medicine*.

[32] Kajihara H, Yamada Y, Shigetomi H, Higashiura Y, Kobayashi H (2012). The dichotomy in the histogenesis of endometriosis-associated ovarian cancer: clear cell-type versus endometrioid-type adenocarcinoma. *International Journal of Gynecological Pathology*.

[33] Okamoto T, Mandai M, Mtsumura N (2013). Hepatocyte nuclear factor-1*β* (HNF-1*β*) promotes glucose uptake and glycolytic activity in ovarian clear cell carcinoma. *Molecular Carcinogenesis*.

[34] Mandai M, Matsumura N, Baba T, Yamaguchi K, Hamanishi J, Konishi I (2011). Ovarian clear cell carcinoma as a stress-responsive cancer: influence of the microenvironment on the carcinogenesis and cancer phenotype. *Cancer Letters*.

[35] Yamaguchi K, Mandai M, Toyokuni S (2008). Contents of endometriotic cysts, especially the high concentration of free iron, are a possible cause of carcinogenesis in the cysts through the iron-induced persistent oxidative stress. *Clinical Cancer Research*.

[36] Coquelle A, Toledo F, Stern S, Bieth A, Debatisse M (1998). A new role for hypoxia in tumor progression: induction of fragile site triggering genomic rearrangements and formation of complex DMs and HSRs. *Molecular Cell*.

[37] Meng AX, Jalali F, Cuddihy A (2005). Hypoxia down-regulates DNA double strand break repair gene expression in prostate cancer cells. *Radiotherapy and Oncology*.

[38] Gram IT, Lukanova A, Brill I (2012). Cigarette smoking and risk of histological subtypes of epithelial ovarian cancer in the EPIC cohort study. *International Journal of Cancer*.

[39] Mink PJ, Sherman ME, Devesa SS (2002). Incidence patterns of invasive and borderline ovarian tumors among white women and black women in the United States: results from the SEER program, 1978–1998. *Cancer*.

[40] Yamaguchi K, Mandai M, Oura T (2010). Identification of an ovarian clear cell carcinoma gene signature that reflects inherent disease biology and the carcinogenic processes. *Oncogene*.

[41] Matsumura N, Mandai M, Okamoto T (2010). Sorafenib efficacy in ovarian clear cell carcinoma revealed by transcriptome profiling. *Cancer Science*.

[42] Koshiyama M, Matsumura N, Baba T, Yamaguchi K, Yoshioka Y, Konishi I (2013). Two cases of recurrent ovarian clear cell carcinoma treated with sorafenib. *Cancer Biology and Therapy*.

[43] Palacios J, Gamallo C (1998). Mutations in the *β*-catenin gene (CTNNB1) in endometrioid ovarian carcinomas. *Cancer Research*.

[44] Kobayashi H, Kajiwara H, Kanayama S (2009). Molecular pathogenesis of endometriosis-associated clear cell carcinoma of the ovary. Review. *Oncology Reports*.

[45] Campbell IG, Russell SE, Choong DY (2004). Mutation of the PIK3CA gene in ovarian and breast cancer. *Cancer Research*.

[46] Willner J, Wurz K, Allison KH (2007). Alternate molecular genetic pathways in ovarian carcinomas of common histological types. *Human Pathology*.

[47] Fujita M, Enomoto T, Yoshino K (1995). Microsatellite instability and alterations in the hMSH2 gene in human ovarian cancer. *International Journal of Cancer*.

[48] Gras E, Catasus L, Arguelles R (2001). Microsatellite instability, MLH-1 promoter hypermethylation, and frameshift mutations at coding mononucleotide repeat microsatellites in ovarian tumors. *Cancer*.

[49] Czriker M, Dockerty M (1954). Mucinous cystadenomas and mucinous cystadenocarcinomas of the ovary, a clinical and pathological study of 355 cases. *Cancer*.

[50] Woodruff JD, Bie LS, Sherman RJ (1960). Mucinous tumors of the ovary. *Obstetrics and Gynecology*.

[51] Scully RE, Young RH, Clement PB (1998). Tumors of the ovary, maldeveloped gonads, fallopian tube, and broad ligament. *Atlas of Tumor Pathology*.

[52] Feeley KM, Wells M (2001). Precursor lesions of ovarian epithelial malignancy. *Histopathology*.

[53] Resta L, Russo S, Coluci GA, Prat J (1993). Morphologic precursors of ovarian epithelial tumors. *Obstetrics and Gynecology*.

[54] Salazar H, Godwin AK, Daly MB (1996). Microscopic benign and invasive malignant neoplasms and a cancer-prone phenotype in prophylactic oophorectomies. *Journal of the National Cancer Institute*.

[55] Rutgers JL, Scully RE (1988). Ovarian mullerian mucinous papillary cystadenomas of borderline malignancy. A clinicopathologic analysis. *Cancer*.

[56] Lim D, Oliva E (2013). Precursors and pathogenesis of ovarian carcinoma. *Pathology*.

[57] Nomura K, Aizawa S (1997). A histogenetic consideration of ovarian mucinous tumors based on an analysis of lesions associated with teratomas or Brenner tumors. *Pathology International*.

[58] Hoerl HD, Hart WR (1998). Primary ovarian mucinous cystadenocarcinomas. *The American Journal of Surgical Pathology*.

[59] Lee KR, Scully RE (2000). Mucinous tumors of the ovary: a clinicopathologic study of 196 borderline tumors (of intestinal type) and carcinomas, including an evaluation of 11 cases with ‘pseudomyxoma peritonei’. *The American Journal of Surgical Pathology*.

[60] Pejovic T, Bürki N, Odunsi K (1999). Well-differentiated mucinous carcinoma of the ovary and a coexisting brenner tumor both exhibit amplification of 12q14-21 by comparative genomic hybridization. *Gynecologic Oncology*.

[61] Koshiyama M, Konishi I, Yoshida M (1994). Transitional cell carcinoma of the fallopian tube: a light and electron microscopic study. *International Journal of Gynecological Pathology*.

[62] Seidman JD, Khedmati F (2008). Exploring the histogenesis of ovarian mucinous and transitional cell (Brenner) neoplasms and their relationship with walthard cell nests: a study of 120 tumors. *Archives of Pathology and Laboratory Medicine*.

[63] Enomoto T, Weghorst CM, Inoue M, Tanizawa O, Rice JM (1991). K-ras activation occurs frequently in mucinous adenocarcinomas and rarely in other common epithelial tumors of the human ovary. *The American Journal of Pathology*.

[64] Ichikawa Y, Nishida M, Suzuki H (1994). Mutation of K-ras protooncogene is associated with histological subtypes in human mucinous ovarian tumors. *Cancer Research*.

[65] Caduff RF, Svoboda-Newman SM, Ferguson AW, Johnston CM, Frank TS (1999). Comparison of mutations of Ki-RAS and p53 immunoreactivity in borderline and malignant epithelial ovarian tumors. *The American Journal of Surgical Pathology*.

[66] Gemignani ML, Schlaerth AC, Bogomolniy F (2003). Role of KRAS and BRAF gene mutations in mucinous ovarian carcinoma. *Gynecologic Oncology*.

[67] Mok SC, Bell DA, Knapp RC (1993). Mutation of K-ras protooncogene in human ovarian epithelial tumors of borderline malignancy. *Cancer Research*.

[68] Mandai M, Konishi I, Kuroda H (1998). Heterogeneous distribution of K-ras-mutated epithelia in mucinous ovarian tumors with special reference to histopathology. *Human Pathology*.

[69] Ronnett BM, Zahn CM, Kurman RJ, Kass ME, Sugarbaker PH, Shmookler BM (1995). Disseminated peritoneal adenomucinosis and peritoneal mucinous carcinomatosis: a clinicopathologic analysis of 109 cases with emphasis on distinguishing pathologic features, site of origin, prognosis and relationship to ‘pseudomyxoma peritonei’. *The American Journal of Surgical Pathology*.

[70] Guerrieri C, Franlund B, Boeryd B (1995). Expression of cytokeratin 7 in simultaneous mucinous tumors of the ovary and appendix. *Modern Pathology*.

[71] Tenti P, Aguzzi A, Riva C (1992). Ovarian mucinous tumors frequently express markers of gastric, intestinal, and pancreatobiliary epithelial cells. *Cancer*.

[72] Rodriguez IM, Irving JA, Prat J (2004). Endocervical-like mucinous borderline tumors of the ovary: a clinicopathologic analysis of 31 cases. *The American Journal of Surgical Pathology*.

[73] Lee KR, Nucci MR (2003). Ovarian mucinous and mixed epithelial carcinomas of mullerian (endocervical-like) type: a clinicopathologic analysis of four cases of an uncommon variant associated with endometriosis. *International Journal of Gynecological Pathology*.

[74] Jha P, Ranson MK, Nguyen SN, Yach D (2002). Estimates of global and regional smoking prevalence in 1995, by age and sex. *The American Journal of Public Health*.

[75] Jordan SJ, Whiteman DC, Purdie DM, Green AC, Webb PM (2006). Does smoking increase risk of ovarian cancer? A systematic review. *Gynecologic Oncology*.

[76] Benito V, Lubrano A, Arencibia O (2010). Serous and mucinous borderline ovarian tumors: are there real differences between these two entities?. *European Journal of Obstetrics Gynecology and Reproductive Biology*.

[77] Seidman JD, Kurman RJ (1996). Subclassification of serous borderline tumors of the ovary into benign and malignant types: a clinicopathologic study of 65 advanced stage cases. *The American Journal of Surgical Pathology*.

[78] Kurman RJ, Visvanathan K, Roden R, Wu TC, Shih I (2008). Early detection and treatment of ovarian cancer: shifting from early stage to minimal volume of disease based on a new model of carcinogenesis. *The American Journal of Obstetrics and Gynecology*.

[79] Vang R, Shih I, Kurman RJ (2009). Ovarian low-grade and high-grade serous carcinoma: pathogenesis, clinicopathologic and molecular biologic features, and diagnostic problems. *Advances in Anatomic Pathology*.

[80] McCluggage WG (2010). The pathology of and controversial aspects of ovarian borderline tumours. *Current Opinion in Oncology*.

[81] Singer G, Oldt R, Cohen Y (2003). Mutations in BRAF and KRAS characterize the development of low-grade ovarian serous carcinoma. *Journal of the National Cancer Institute*.

[82] Haas CJ, Diebold J, Hirschmann A, Rohrbach H, Löhrs U (1999). In serous ovarian neoplasms the frequency of Ki-ras mutations correlates with their malignant potential. *Virchows Archiv*.

[83] Seiben NL, Macropoulos P, Roemen GM (2004). In ovarian neoplasms, BRAF, but not KRAS, mutations are restricted to low-grade serous tumours. *Journal of Pathology*.

[84] Mayr D, Hirschmann A, Löhrs U, Diebold J (2006). KRAS and BRAF mutations in ovarian tumors: a comprehensive study of invasive carcinomas, borderline tumors and extraovarian implants. *Gynecologic Oncology*.

[85] Ho C-L, Kurman RJ, Dehari R, Wang T, Shih I (2004). Mutations of BRAF and KRAS precede the development of ovarian serous borderline tumors. *Cancer Research*.

[86] Singer G, Stöhr R, Cope L (2005). Patterns of p53 mutations separate ovarian serous borderline tumors and low- and high-grade carcinomas and provide support for a new model of ovarian carcinogenesis: a mutational analysis with immunohistochemical correlation. *The American Journal of Surgical Pathology*.

[87] Dehari R, Kurman RJ, Logani S, Shih I (2007). The development of high-grade serous carcinoma from atypical proliferative (borderline) serous tumors and low-grade micropapillary serous carcinoma: a morphologic and molecular genetic analysis. *The American Journal of Surgical Pathology*.

[88] Pradhan M, Davidson B, Tropé CG, Danielsen HE, Abeler VM, Risberg B (2009). Gross genomic alterations differ between serous borderline tumors and serous adenocarcinomas-an image cytometric DNA ploidy analysis of 307 cases with histogenetic implications. *Virchows Archiv*.

[89] Kuo K-T, Guan B, Feng Y (2009). Analysis of DNA copy number alterations in ovarian serous tumors identifies new molecular genetic changes in low-grade and high-grade carcinomas. *Cancer Research*.

[90] Jones S, Wang T-L, Kurman RJ (2012). Low-grade serous carcinomas of the ovary contain very few point mutations. *Journal of Pathology*.

[91] Boyd J, Luo B, Peri S (2013). Whole exome sequence analysis of serous borderline tumors of the ovary. *Gynecologic Oncology*.

[92] The Cancer Genome Atlas Network (2011). Integrated genomic analyses of ovarian carcinoma. *Nature*.

[93] Lauchlan SC (1972). The secondary Mullerian system. *Obstetrical and Gynecological Survey*.

[94] Dubeau L (2008). The cell of origin of ovarian epithelial tumours. *The Lancet Oncology*.

[95] Altaras MM, Jaffe R, Corduba M, Holtzinger M, Bahary C (1990). Primary paraovarian cystadenocarcinoma: clinical and management aspects and literature review. *Gynecologic Oncology*.

[96] de Areia AL, Frutuoso C, Amaral N, Dias I, De Oliveira C (2004). Paraovarian tumor of borderline malignancy—a case report. *International Journal of Gynecological Cancer*.

[97] Kurman RJ, Vang R, Junge J, Hannibal CG, Kjaer SK, Shih I (2011). Papillary tubal hyperplasia: the putative precursor of ovarian atypical proliferative (borderline) serous tumors, noninvasive implants, and endosalpingiosis. *The American Journal of Surgical Pathology*.

[98] Li J, Abushahin N, Pang S (2011). Tubal origin of “ovarian” low-grade serous carcinoma. *Modern Pathology*.

[99] Christie M, Oehler MK (2006). Molecular pathology of epithelial ovarian cancer. *Journal of the British Menopause Society*.

[100] Powell BC, Kenley E, Chen LM (2005). Risk-reducing salpingo-oophorectomy in BRCA mutation carriers: role of serial sectioning in the detection of occult malignancy. *Journal of Clinical Oncology*.

[101] Finch A, Shaw P, Rosen B, Murphy J, Narod SA, Colgan TJ (2006). Clinical and pathologic findings of prophylactic salpingo-oophorectomies in 159 BRCA1 and BRCA2 carriers. *Gynecologic Oncology*.

[102] Kindelberger DW, Lee Y, Miron A (2007). Intraepithelial carcinoma of the fimbria and pelvic serous carcinoma: evidence for a causal relationship. *The American Journal of Surgical Pathology*.

[103] Callahan MJ, Crum CP, Medeiros F (2007). Primary fallopian tube malignancies in BRCA-positive women undergoing surgery for ovarian cancer risk reduction. *Journal of Clinical Oncology*.

[104] Folkins AK, Jarboe EA, Saleemuddin A (2008). A candidate precursor to pelvic serous cancer (p53 signature) and its prevalence in ovaries and fallopian tubes from women with BRCA mutations. *Gynecologic Oncology*.

[105] Piek JMJ, Verheijen RHM, Kenemans P, Massuger LF, Bulten H, van Diest PJ (2003). BRCA1/2-related ovarian cancers are of tubal origin: a hypothesis. *Gynecologic Oncology*.

[106] Chen EY, Mehra K, Mehrad M (2010). Secretory cell outgrowth, PAX2 and serous carcinogenesis in the fallopian tube. *Journal of Pathology*.

[107] Chene G, Dauplat J, Radosevic-Robin N, Cayre A, Penault-Llorca F (2013). Tu-be or not tu-be: that is the question… about serous ovarian carcinogenesis. *Critical Reviews in Oncology/Hematology*.

[108] Tung CS, Mok SC, Tsang YT (2009). PAX2 expression in low malignant potential ovarian tumors and low-grade ovarian serous carcinomas. *Modern Pathology*.

[109] Roh MH, Yassin Y, Miron A (2010). High-grade fimbrial-ovarian carcinomas are unified by altered p53, PTEN and PAX2 expression. *Modern Pathology*.

[110] Rabban JT, McAlhany S, Lerwill MF, Grenert JP, Zaloudek CJ (2010). PAX2 distinguishes benign mesonephric and mullerian glandular lesions of the cervix from endocervical adenocarcinoma, including minimal deviation adenocarcinoma. *The American Journal of Surgical Pathology*.

[111] Visvanathan K, Gross AL, Kurman RJ, Vang R, Shih I (2010). Precursor lesions of high-grade serous ovarian carcinoma: morphological and molecular characteristics. *Journal of Oncology*.

[112] Carcangiu ML, Radice P, Manoukian S (2004). Atypical epithelial proliferation in fallopian tubes in prophylactic salpingo-oophorectomy specimens from BRCA1 and BRCA2 germline mutation carriers. *International Journal of Gynecological Pathology*.

[113] Mehrad M, Ning G, Chen EY, Mehra KK, Crum CP (2010). A pathologist’s road map to benign, precancerous, and malignant intraepithelial proliferations in the fallopian tube. *Advances in Anatomic Pathology*.

[114] Jarboe EA, Folkins AK, Drapkin R, Ince TA, Agoston ES, Crum CP (2008). Tubal and ovarian pathways to pelvic epithelial cancer: a pathological perspective. *Histopathology*.

[115] Salvador S, Rempel A, Soslow RA, Gilks B, Huntsman D, Miller D (2008). Chromosomal instability in fallopian tube precursor lesions of serous carcinoma and frequent monoclonality of synchronous ovarian and fallopian tube mucosal serous carcinoma. *Gynecologic Oncology*.

[116] Seidman JD, Yemelyanova A, Zaino RJ, Kurman RJ (2011). The fallopian tube-peritoneal junction: a potential site of carcinogenesis. *International Journal of Gynecological Pathology*.

[117] Kuhn E, Meeker A, Wang TL, Sehdev AS, Kurman RJ, Shih I (2010). Shortened telomeres in serous tubal intraepithelial carcinoma: an early event in ovarian high-grade serous carcinogenesis. *The American Journal of Surgical Pathology*.

[118] Gorgoulis VG, Vassiliou LF, Karakaidos P (2005). Activation of the DNA damage checkpoint and genomic instability in human precancerous lesions. *Nature*.

[119] Chene G, Rahimi K, Mes-Masson AM, Provencher D (2013). Surgical implications of the potential new tubal pathway for ovarian carcinogenesis. *Journal of Minimally Invasive Gynecology*.

[120] Bell DA, Scully RE (1994). Early de novo ovarian carcinoma: a study of fourteen cases. *Cancer*.

[121] Flesken-Nikitin A, Hwang CI, Cheng CY, Michurina TV, Enikolopov G, Nikitin AY (2013). Ovarian surface epithelium at the junction area contains a cancer-prone stem cell niche. *Nature*.

